# Dose selection method for pharmacokinetic study in hemodialysis patients using a subpharmacological dose: oseltamivir as a model drug

**DOI:** 10.1186/1471-2369-15-46

**Published:** 2014-03-17

**Authors:** Dong Ki Kim, Jay Wook Lee, Kwang-Hee Shin, Sejoong Kim, Kook-Hwan Oh, Myounghee Kim, Kyung-Sang Yu, Jung Pyo Lee, Chun-Soo Lim, Yon Su Kim, Kwon Wook Joo

**Affiliations:** 1 Department of Internal Medicine, Seoul National University College of Medicine, Seoul, Korea; 2 Department of Internal Medicine, Chung-Ang University College of Medicine, Seoul, Korea; 3 College of Pharmacy, Kyungpook National University, Daegu, Korea; 4 Department of Internal Medicine, Seoul National University Bundang Hospital, Seongnam, Korea; 5 Department of Dental Hygiene, College of Health Science, Eulji University, Seongnam, Korea; 6 Department of Clinical Pharmacology and Therapeutics, Seoul National University College of Medicine, Seoul, Korea; 7 Department of Internal Medicine, Seoul National University Boramae Medical Center, Seoul, Korea; 8 Department of Internal Medicine, Seoul National University Hospital, 101 Daehak-ro, Jongro-gu, Seoul 110-744, Korea

**Keywords:** Hemodialysis, Pharmacokinetics, Drug, Dosage

## Abstract

**Background:**

Dose selection is an important step in pharmacokinetic (PK) studies of hemodialysis patients. We propose a simulation-based dose-selection method for PK studies of hemodialysis patients using a subpharmacological dose of oseltamivir as a model drug.

**Methods:**

The concentrations of oseltamivir and its active metabolite, oseltamivir carboxylate (OC), were measured by liquid chromatography-tandem mass spectrometry. To determine a low oseltamivir dose exhibiting PK linearity, a pilot low dose determination investigation (n = 4) was performed using a single administration dose-escalation study. After the dose was determined, a low dose study (n = 10) was performed, and the optimal dose required to reach the hypothetical target OC exposure (area under the concentration-time curve [AUC] of 60,000 ng · hr/mL) was simulated using a nonparametric superposition method. Finally, observed PKs at the optimal dose were compared to the simulated PKs to verify PK predictability.

**Results:**

In the pilot low dose determination study, 2.5 mg of oseltamivir was determined to be the low dose. Subsequently, we performed a single-dose PK study with the low oseltamivir dose in an additional group of 10 hemodialysis patients. The predicted AUC_last_ of OC following continuous oseltamivir doses was simulated, and 35 mg of oseltamivir corresponded to the hypothetical target AUC_last_ of OC. The observed PK profiles of OC at a 35-mg oseltamivir dose and the simulated data based on the low dose study were in close alignment.

**Conclusion:**

The results indicate that the proposed method provides a rational approach to determine the proper PK dose in hemodialysis patients.

## Background

In addition to impaired renal function, hemodialysis patients may also possess altered pharmacokinetics (PKs) caused by hemodialysis itself. Hemodialysis is an important drug elimination rout, which is influenced by the characteristics of the drug, dialysis membrane and dose of dialysis [1]. In addition, hemodialysis may influence the metabolic clearance of a drug by affecting drug-metabolizing enzymes or transporters [2]. However, despite the difficulty of predicting PKs, which results from various interfering factors, previous PK studies have often excluded the hemodialysis patient population [3,4]. One reason for frequent exclusion is the uncertainty of selecting proper dosing for a PK study. In fact, improper dosing during PK studies can cause several problems. For example, administration of a potentially unsafe high dose during a PK study may lead to drug accumulation and adverse drug reactions, particularly in hemodialysis patients. Conversely, administering a dose that is too low may lead to unnecessary PK study repetition. Therefore, our objective was to develop a dose selection method for PK studies in hemodialysis patients using the simulation-extrapolation of an optimal PK dose from the PK parameters of a subpharmacological dose.

In this study, we chose oseltamivir as a model drug to evaluate this proposed method because PKs of its active metabolite, oseltamivir carboxylate (OC), were dramatically altered, especially in patients undergoing hemodialysis [5]. Orally administered oseltamivir is rapidly converted into OC by first pass metabolism, which is minimally affected by liver function [6]. Consequently, approximately 80% of orally administered oseltamivir reaches the systemic circulation as OC, and plasma OC concentrations exhibit minimal inter- and intra-subject variability [7]. The excretion of OC occurs exclusively via the kidneys, and therefore, OC exposure is substantially increased in patients with end-stage renal disease (ESRD) [7,8]. In addition, hemodialysis may also affect the PKs of oseltamivir because a significant fraction of OC is removed by hemodialysis [5,7]. Moreover, clinical concerns have arisen regarding oseltamivir use in patients with ESRD because of their high mortality from influenza, generally severe courses of influenza infection, and reduced response to vaccinations [9]. These PK and clinical characteristics of oseltamivir correspond to a drug category where the U.S. FDA recommends mandatory PK studies in patients with impaired renal function [10]. Therefore, we selected oseltamivir, even though this drug is already approved for mass marketing and its therapeutic dose PKs in ESRD patients are well known [5].

## Methods

This study was conducted at the Clinical Trials Center and Dialysis Unit of Seoul National University Hospital (Seoul, Korea) from March 2011 to March 2012. The Institutional Review Board and the Korean Food and Drug Administration approved the study protocol. All procedures were performed in accordance with the recommendations of the Declaration of Helsinki. All subjects provided written informed consent and received prorated compensation after the study.

### Study subjects

Eligibility criteria were an age >20 years and anuric non-diabetic ESRD undergoing intermittent hemodialysis. Exclusion criteria included current pregnancy or lactation, the prior use of an antiviral agent within the past three months, a history of allergic reaction to oseltamivir, alcohol and/or drug abuse, and participation in any other clinical study within two months prior to the present study. Additionally, subjects with gastrointestinal disease that could alter drug absorption were also excluded from the study. All other medications except anti-hypertensives were prohibited from three days prior to oseltamivir administration to the end of the study. As a safety precaution, subjects underwent a screening evaluation, including a medical history, vital signs, 12-lead electrocardiography, and clinical laboratory tests (complete blood count and serum chemistry profiles) that were performed within 4 weeks of oseltamivir administration. Subjects were fasted 8 hr prior and 4 hr following oseltamivir administration. During the admission period, subjects received and ate only the standard food and drink provided by the clinical trials center and were not allowed to consume fruit juice or any beverage containing alcohol or caffeine. All observed or self-reported adverse events that occurred over the study period were recorded. Blood pressure, pulse rate, and body temperature were measured at each sampling time.

### Study design

#### Step 1: pilot low dose determination study

The dose-finding step was designed as an open-label, single sequence dose escalation, single-dosing study to determine the lowest oseltamivir dose detectable in plasma by liquid chromatography-tandem mass spectrometry (LC-MS/MS) and showing dose-linearity with two higher oseltamivir doses. A total of four hemodialysis patients were enrolled in this pilot study. Considering the lower limit of quantification (LLOQ) for plasma oseltamivir concentrations (0.2 ng/mL) by LC-MS/MS as well as the oseltamivir PKs in patients with advanced renal dysfunction not on dialysis [7], a 2.5-mg oseltamivir dose was expected as the lowest detectable dose. The PK studies were performed using 3-week wash-out periods with dose doubling until the PK parameters at certain doses exhibited dose-linearity with the PK parameters of two higher doses. All procedures were identical in all periods except for the dose administered. All patients were dialyzed for 4 hr using a high-flux membrane (Polyflux 170H; Gambro, Lund, Sweden; ultrafiltration coefficient, 70 mL/hr/Kg; single use). Blood flow was maintained at a constant rate of 250 mL/min, and the dialysate flow rate was also held constant at 500 mL/min.

#### Step 2: low dose PK study and extrapolation of the appropriate PK dose

After determining the low oseltamivir dose during the pilot study, an additional set of 10 hemodialysis patients were evaluated in a single dose standard PK study using the low oseltamivir dose. Subsequently, the predicted area under the concentration-time curve (AUC) from drug administration to 72 hr post-administration or to the last measurable time point (AUC_last_) of OC following continuous doses up to 150 mg was simulated from the low dose data using a nonparametric superposition method with the WinNolin® version 6.3 software (Pharsight Corporation, Mountain View, CA, USA). Based on the results of a previous oseltamivir PK study in hemodialysis patients [5], a hypothetical target AUC_last_ of OC was defined as 60,000 ng · hr/mL, and the oseltamivir dose required to reach this target was considered to be the optimal PK dose.

#### Step 3: method validation

To validate the dose-selection method, the same participants were evaluated in a PK study using the extrapolated dose after a 3-week wash-out period. The observed PKs were compared to the simulated PKs based on the low dose study. The simulations for plasma oseltamivir, plasma OC, and dialysate OC after the administration of the extrapolated oseltamivir dose were performed using a nonparametric superposition method with the WinNolin® version 6.3 software.

### PK assessment

The study subjects received a solution containing oseltamivir dissolved in 20 mL of purified water followed by the administration of 150 mL of water immediately after the completion of hemodialysis. Blood samples were collected before oseltamivir administration and at 1, 1.5, 2, 2.5, 3, 6, 12, 24, 44 (dialysis start), 48 (dialysis end), and 72 hr after oseltamivir administration through an indwelling catheter or direct venipuncture into pre-cooled fluoride tubes (BD, Franklin Lakes, NJ, USA). Plasma was separated from blood by centrifugation at 4°C within 2 hr of collection. Dialysate was also collected hourly during the dialysis. All samples were stored at -70°C until further use.

### Determination of plasma and dialysate oseltamivir and OC concentrations

Oseltamivir, OC, and their trideuterated species used as internal standards were kindly provided by Hoffmann-La Roche (Basel, Switzerland). The quantification of plasma and dialysate oseltamivir and OC were performed using an Agilent 6460 triple quadrupole mass spectrometer (Palo Alto, CA, USA) coupled to an Agilent 1260 LC system (Palo Alto) using oseltamivir d3-carboxylate as an internal standard. The LLOQ were determined from standard curves of oseltamivir (0.2-100 ng/ml) and OC (2–500 ng/mL). The LLOQ was 0.2 ng/ml for oseltamivir and 2 ng/mL for OC. The coefficients of determination of the calibration curves were all >0.99. Standard curves were accurate (93.7% ≤% accuracy ≤101.2%) and precise (0.775% ≤% precision ≤6.924%) across a range of 0.5 to 80 ng/mL for oseltamivir and were accurate (98.8% ≤% accuracy ≤103.5%) and precise (1.032% ≤% precision ≤2.857%) across a range of 5 to 400 ng/mL for OC.

### PK and statistical analyses

The PK parameters were calculated by a non-compartmental method using Phoenix®. The AUC_last_ was computed using the linear trapezoidal approximation method. The maximum observed plasma concentration (C_max_) and time to reach C_max_ (T_max_) were directly determined from the individual concentration–time profiles. The oral plasma clearance (CL/F) was calculated as the dose/AUC_last_. Intradialytic oseltamivir and OC clearance were calculated using the recovery method and the following equation: CL_HD_ = R/AUC_HD_, where CL_HD_ is the oseltamivir or OC clearance through hemodialysis, R is the amount of oseltamivir or OC recovered during the dialysis session, and AUC_HD_ is the area under the serum concentration-time curve during hemodialysis.

The Kruskal-Wallis test was used to compare the dose-normalized C_max_ and AUC_last_ between doses of oseltamivir. The dose proportionality in the dose-finding pilot study was tested using a power model analysis of log-transformed PK parameters versus the log-transformed dose. Dose proportionality was assumed if the slope was not statistically significantly different from unity and the 95% confidence interval (CI) included 1.0 [11]. The extrapolated dose of oseltamivir was simulated using nonparametric superposition (WinNolin® version 6.3 software), which can be used to predict drug concentrations after multiple dosing at steady state, based on non-compartmental results of single dose data and not assuming any PK model [12].

For PK data, summary statistics are presented as the means (SD). SPSS (version 12.0; SPSS Korea, Seoul, Korea) was used for the statistical analyses, and a *P* value of less than 0.05 was considered statistically significant.

## Results

### Subjects

The demographic characteristics of the study participants are listed in Table 1. No subjects exhibited adverse events after oseltamivir administration in the dose-finding pilot study. However, two of the 10 participants who received a dose of 35 mg of oseltamivir experienced nausea, which was mild in intensity. No serious adverse events occurred during the study period.

**Table 1:** Demographic data for study subjects

	**Low dose determination pilot study (n = 4)**	**Low dose study and method validation (n = 10)**
Age (yr)	56.8 (14.8)	52.4 (14.2)
Sex (male: female)	2:2	6:4
Dry weight (Kg)	50.3 (13.4)	52.9 (12.1)
Body mass index (kg/m^2^)	20.6 (5.0)	20.1 (3.0)
Cause of ESRD (n)		
Chronic glomerulonephritis	2	4
Hypertension	1	3
Unknown	1	3
Time on hemodialysis	3.3 (0.8)	4.1 (1.6)
Hemoglobin (g/dL)	10.50 (0.58)	10.88 (0.92)
Dialysis efficiency		
(at 44–48 hr of study period)		
Single-pool Kt/V_Urea_	1.47 (0.10)	1.58 (0.28)
Urea reduction ration (%)	70.7 (2.1)	73.5 (5.3)
Ultrafiltration rate (L/session)	2.60 (0.13)	2.10 (0.76)

Data presented mean (standard deviation) or numbers.

### Pharmacokinetics

#### Pilot low dose determination study

**Figure 1:**
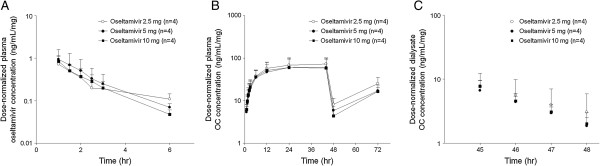
**Semi-log plots of the dose-normalized mean plasma oseltamivir (A), plasma oseltamivir carboxylate (B), and dialysate oseltamivir carboxylate (C) concentrations following a single oral dose of 2.5 (○), 5.0 (●), and 10.0 mg (■) of oseltamivir.** Error bars represent the standard deviation. OC, oseltamivir carboxylate.

Figure 1 shows a semi-log plot of the mean plasma oseltamivir, plasma OC, and dialysate OC concentrations normalized to a 1-mg oseltamivir dose versus time post administration of a single oral oseltamivir dose of 2.5, 5.0, and 10.0 mg. Although oseltamivir could not be detected after 6 hr, the shapes of the oseltamivir and OC dose response curves were similar, and the curves were predominantly superimposable at the various doses. Similarly, the mean AUC_last_ values of oseltamivir and OC, and the AUC_HD_ of OC increased in a dose-dependent manner (Table 2). Additionally, no differences were detected between groups with respect to the dose-normalized C_max_ and AUC_last_ using the Kruskal-Wallis test (Table 3). Furthermore, power model analysis displayed dose-proportional increases in the AUC_last_ and C_max_ of oseltamivir and OC within a dose range of 2.5 to 10 mg. The slope estimates were close to unity for the AUC_last_ and C_max_ of oseltamivir and OC and AUC_HD_ of OC. All the corresponding 95% CIs included 1.0 (Table 4).

**Table 2:** Pharmacokinetic parameters of oseltamivir and oseltamivir carboxylate following a single oral administration of 2.5, 5, and 10 mg of oseltamivir

**Parameter**	**Oseltamivir**			**Oseltamivir carboxylate**		
	**Oseltamivir 2.5 mg**	**Oseltamivir 5 mg**	**Oseltamivir 10 mg**	**Oseltamivir 2.5 mg**	**Oseltamivir 5 mg**	**Oseltamivir 10 mg**
C_max_ (ng/mL)	1.81 (0.92)	4.68 (3.28)	8.40 (3.03)	181.07 (96.38)	410.35 (206.54)	599.23 (360.64)
T_max_ (hr)	1.00 [1.00, 1.55]	1.06 [1.00, 1.13]	1.00 [1.00, 1.55]	44.11 [24.00, 44.25]	43.99 [24.0, 44.05]	24.01 [24.00, 43.68]
AUC_last_ (ng h/mL)	3.24 (0.81)	9.60 (6.83)	15.47 (5.48)	7697.12 (3930.52)	12497.57 (3537.45)	25394.42 (12912.41)
AUC_HD_ (ng h/mL)	NA	NA	NA	288.52 (119.78)	458.82 (226.88)	784.87 (205.39)
CL/F (L/hr)	554.41 (37.86)	558.27 (252.14)	645.73 (161.19)	NA	NA	NA
CL_HD_ (mL/hr)	NA	NA	NA	5.14 (1.87)	6.41 (3.43)	6.92 (1.30)
Drug excreted by dialysis (%)^*^	NA	NA	NA	0.06 (0.04)	0.05 (0.02)	0.04 (0.01)

Data presented mean (SD) except T_max_, median [min, max]; C_max_, peak plasma concentration; T_max_, time to C_max_; AUC, area under the plasma concentration–time curve; CL/F, oral plasma clearance; CL_HD_, dialysis clearance; NA, not applicable; ^*^amount excreted of oseltamivir carboxylate / administered oseltamivir.

**Table 3:** **Dose-normalized C**_**max **_**and AUC**_**last **_**following a single oral administration of 2.5, 5, and 10 mg of oseltamivir**

**Parameter**	**Oseltamivir**			*P*-value^*^	**Oseltamivir carboxylate**			*P*-value^*^
	**Oseltamivir 2.5 mg (n = 4)**	**Oseltamivir 5 mg (n = 4)**	**Oseltamivir 10 mg (n = 4)**		**Oseltamivir 2.5 mg (n = 4)**	**Oseltamivir 5 mg (n = 4)**	**Oseltamivir 10 mg (n = 4)**	
C_max_/dose (ng/mL/mg)	0.72 (0.37)	0.94 (0.66)	0.84 (0.30)	0.981	72.43 (38.55)	82.07 (41.31)	59.92 (36.06)	0.926
AUC_last_/dose (ng · h/mL/mg)	1.29 (0.33)	1.92 (1.37)	1.55 (0.55)	0.981	3078.9 (1572.2)	2499.5 (707.5)	2539.4 (1291.2)	0.944

Data presented mean (SD); C_max_, peak plasma concentration; AUC, area under the plasma concentration–time curve; ^*^The Kruskal-Wallis test.

**Table 4:** Power model analysis for dose-concentration relationship

**Parameters**	**Estimate of β**	**95% CI**
Oseltamivir		
C_max_ (ng/mL)	1.187	0.556-1.818
AUC_last_ (ng h/mL)	1.121	0.652-1.591
Oseltamivir carboxylate		
C_max_ (ng/mL)	0.889	0.232-1.547
AUC_last_ (ng h/mL)	0.897	0.374-1.421
AUC_HD_ (ng h/mL)	1.062	0.507-1.617

C_max_, peak plasma concentration; CI, confidence interval; AUC, area under the plasma concentration–time curve.

Together, the dose relationships of AUC_last_ and C_max_ for oseltamivir and OC within the 2.5 to 10 mg dose range did not deviate from dose proportionality. Accordingly, the subsequent PK study was performed with an oral dose of 2.5 mg of oseltamivir.

#### Low dose PK study and extrapolation of the appropriate PK dose

**Figure 2:**
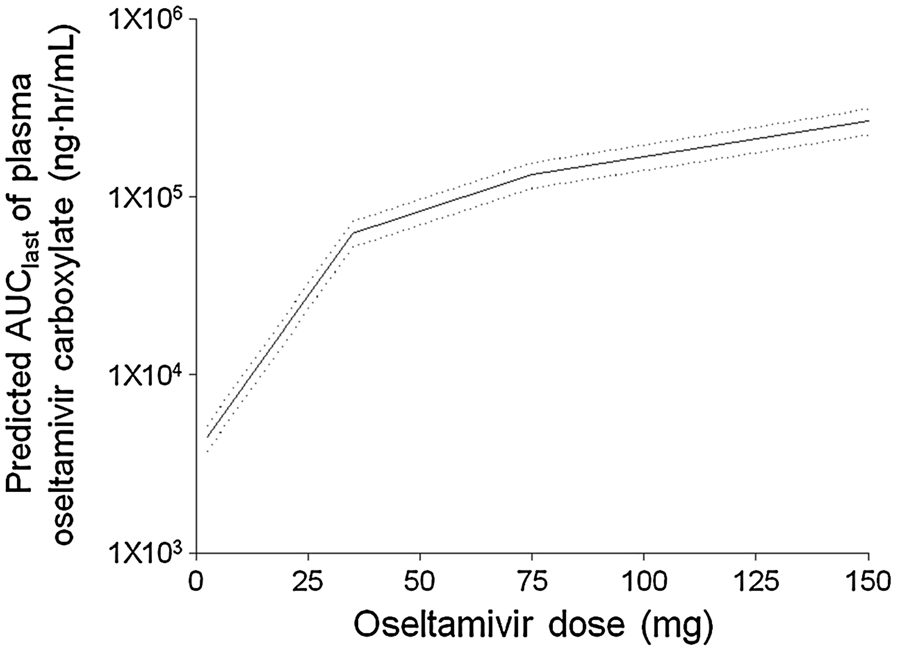
**Predicted AUC**_**last **_**of plasma oseltamivir carboxylate following continuous doses simulated from the low dose data with a nonparametric superposition method.** The solid and dotted lines represent the fitted line and standard error, respectively. OC, oseltamivir carboxylate.

After a low oseltamivir dose of 2.5 mg was determined, we performed a single-dosing standard PK study following the administration of 2.5 mg of oseltamivir with 10 additional hemodialysis patients. The mean C_max_ of oseltamivir and OC were 1.63 ± 0.87 and 128.14 ± 50.41 ng/mL, respectively, and the median T_max_ of oseltamivir and OC were 1.00 (0.98-1.00) and 33.95 (12.00-44.08) hr, respectively. The AUC_last_ values of oseltamivir and OC were 2.55 ± 1.17 and 5604.11 ± 2097.22 ng · hr/mL, respectively. The CL/F of oseltamivir was 618.84 ± 424.92 L/hr, and the CL_HD_ of OC was 7.07 ± 4.30 mL/hr. Subsequently, the predicted AUC_last_ values of OC following continuous doses were simulated from the low dose data using a nonparametric superposition method (Figure 2). The appropriate oral PK oseltamivir dose was determined to be 34.61 mg, which corresponded to the hypothetical target AUC_last_ of 60,000 ng · hr/mL.

#### Method validation

**Figure 3:**
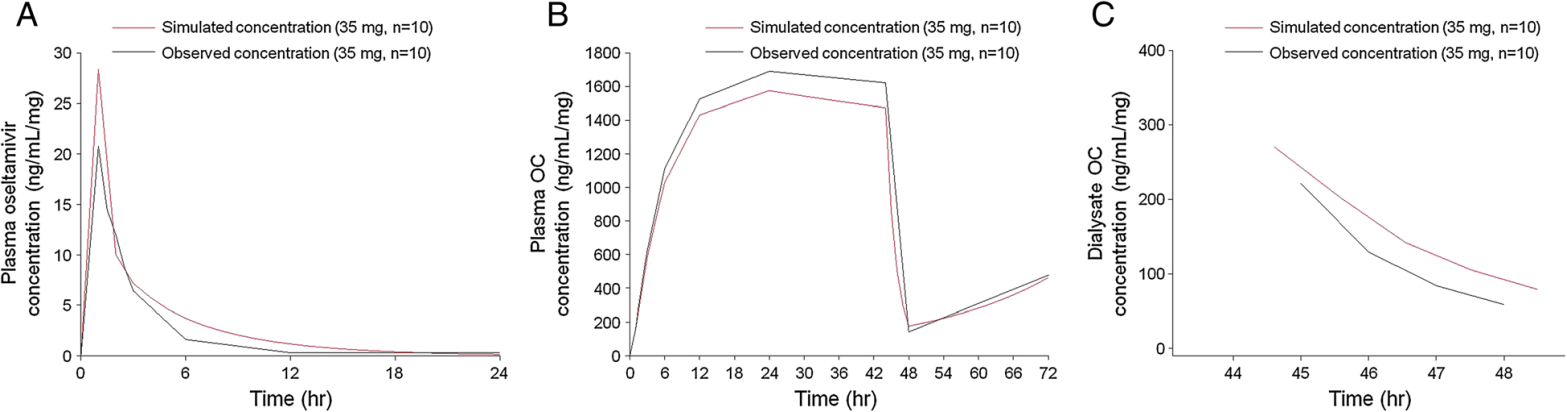
**Simulated (red) and observed (black) concentration-time profiles of plasma oseltamivir (A), plasma oseltamivir carboxylate (B), and dialysate oseltamivir carboxylate (C) concentrations.** OC, oseltamivir carboxylate.

The comparisons of the observed concentration–time profiles of plasma oseltamivir, plasma OC, and dialysate OC at 35 mg of oseltamivir (rounded up from 34.61 mg) and the simulated data based on the PK parameters at a dose of 2.5 mg are shown in Figure 3. The simulated curves correspond closely to the measured values, indicating that low dose PK studies can be used to predict the proper PK dose in hemodialysis patients.

## Discussion

Although current guidelines recommend conducting a PK study for all investigational drugs that are likely be used in ESRD patients [10,13-15], only a small proportion of drugs have been evaluated for PKs in this population. For example, fewer than 30% of the new molecular entities approved by the U.S. FDA between 2003 and 2007 have had their PK profiles assessed in hemodialysis patients during drug development [4]. Moreover, PK studies evaluating drug efficacy and safety, which are usually conducted in phase 3 clinical trials, frequently exclude the hemodialysis patient population [3]. Accordingly, this paucity of PK studies may lead to non-optimized pharmacotherapies and medication-related problems, including drug dosing errors and inadequate efficacy [16]. In addition, the majority of the previously published PK data potentially underestimate the effects of dialysis on drug elimination because recently introduced dialyzers with high permeability and large surface areas were not examined [17-20]. Therefore, PK analyses for many drugs, some of which are already marketed, are necessary to optimize drug dosing in hemodialysis patients.

Individualized dosing recommendations that account for renal function can be derived from the estimation of PKs either through a simulation from detailed PK analyses in patients with varying degrees of renal function or a mixed-effect modeling approach using population-based PK parameters obtained in large clinical studies [1]. However, given the impact of hemodialysis on drug PKs, PK profiles in this patient population cannot simply be extrapolated from the data of pre-dialysis patients with kidney disease [3]. Indeed, hemodialysis may not only eliminate a drug or its active metabolites but can also alter the PK through increased metabolic clearance through the dialysis-induced removal of endogenous inhibitors of metabolic enzymes [2]. Additionally, hemodialysis may decrease the intercompartmental drug clearance [19]. Therefore, standard PK studies in hemodialysis patients should be mandatory because of these dialysis-associated PK perturbations.

Dose selection in PK studies for hemodialysis patients may be an important practical step, and currently, U.S. FDA guidelines recommend using the same dose for all patients in renal impairment-PK studies regardless of the degree of renal function, because the peak concentration is not substantially affected by renal function [10]. However, in case of drugs with extensive renal excretion, such as oseltamivir, exposure to a drug or its active metabolites may be increased more than 10-fold in patients with severe renal impairment when an unadjusted dose is administered [7]. Moreover, approximately 70% of hemodialysis patients lose residual renal function within 1 year after the initiation of hemodialysis [21] and thus the majority of hemodialysis patients do not have residual renal function. Therefore, drug accumulation and adverse drug reactions during PK studies may be more prominent in these patients. In contrast, the selection of a sub-efficacious dose may cause unnecessary repetitious PK studies, thereby increasing study cost and duration. Therefore, a rational approach for dose selection that considers both safety and efficacy should precede the standard PK study, especially for drugs with renal excretion and low therapeutic range. In the present study, we employed oseltamivir as a model drug and demonstrated that PK profiles at therapeutic doses may be predicted using PK modeling and simulation based on low dose PK data. This finding suggests that low dose PK studies are useful in determining the dose used in detailed PK studies in hemodialysis patients. However, considerable debate exists regarding the prediction of the optimal PK dose from low dose studies because of the possibility of PK non-linearity between the doses. Indeed, the extrapolated prediction of the clinical PK parameters from a low dose study may not be accurate if a drug exhibits dose-dependent non-linear PKs [22]. To minimize the non-linearity concern, we performed a dose-escalation PK study in a small number of patients to select a low dose that exhibits linear PKs with two higher doses.

Although micro-dosing PK studies using less than 100 μg of a drug can avoid toxicological effects [23], this approach requires expensive and specialized methodologies, such as positron emission tomography and accelerator mass spectrometry that require radioactive carbon-labeled drugs [24]. In addition, although many comparative studies for various drugs have shown PK linearity between the micro-dose and therapeutic dose [25-28], micro-dosing studies may not always accurately predict the PK parameters at higher therapeutic doses [29-31]. This non-linearity may arise from the non-linear disposition of a drug as well as the saturation of metabolic enzymes or drug transporters [32,33]. Therefore, the valid prediction of pharmacokinetics at therapeutic doses may be further complicated in micro-dosing studies in ESRD patients because impaired drug metabolism and dysfunction of drug transporters in patients with ESRD can alter non-renal clearance of drugs [34,35]. Thus, PK studies using non-radio-labeled drugs at low doses exhibiting linear pharmacokinetics with higher doses may present an alternative and simpler method to determine the PKs of a drug while avoiding unsafe higher doses.

The present study has several limitations that need to be addressed. First, the inter-compartmental clearance was not assessed. Second, the characteristics of OC rebound after dialysis were not fully assessed because no samples were collected after 48 hr. To evaluate the rebound quantitatively, further study should be conducted in HD patients using extended sampling time points.

## Conclusions

In conclusion, the present study suggests that the proper PK drug dose for hemodialysis patients can be simulated from the PK parameters generated after the administration of a low dose at which adverse drug reactions are avoided.

## Abbreviations

AUC: Area under the concentration-time curve; CL: Clearance; Cmax: Maximum observed plasma concentration; ESRD: End-stage renal disease; LC-MS/MS: Liquid chromatography-tandem mass spectrometry; OC: Oseltamivir carboxylate; PK: Pharmacokinetic; Tmax: Time to reach maximum observed plasma concentration.

## Competing interests

The authors declare that they have no competing interests.

## Authors’ contributions

All authors contributed extensively to the work presented in this paper at all stage. DKK, JWL and KWJ conceived the design of this research and wrote the manuscript. KWJ supervised this study. SK, KHO, JPL, CSL, and YSK assembled input data. MK, KSY and KHS performed statistical analyses. JWL and DKK interpreted the data analyses. KWJ gave conceptual advice and commented on the manuscript. All authors read and approved the final manuscript.

## Pre-publication history

The pre-publication history for this paper can be accessed here:

http://www.biomedcentral.com/1471-2369/15/46/prepub
